# Nootropic activity of Crataeva nurvala Buch-Ham against scopolamine induced cognitive impairment

**DOI:** 10.17179/excli2014-541

**Published:** 2015-02-27

**Authors:** Atanu Bhattacharjee, Shastry Chakrakodi Shashidhara, Santanu Saha

**Affiliations:** 1 NGSM Institute of Pharmaceutical Sciences, Department of Pharmacognosy, Deralakatte, Mangalore - 574 018, Karnataka, India; 2 NGSM Institute of Pharmaceutical Sciences, Department of Pharmacology, Deralakatte, Mangalore - 574 018, Karnataka, India

**Keywords:** Crataeva nurvala, nootropic, acetyl cholinesterase

## Abstract

Loss of cognition is one of the age related mental problems and a characteristic symptom of neurodegenerative disorders like Alzheimer’s. *Crataeva nurvala* Buch-Ham, a well explored traditional Indian medicinal plant of Westernghats, is routinely used as folkloric medicine to treat various ailments in particular urolithiasis and neurological disorders associated with cognitive dysfunction. The objective of the study was to evaluate the nootropic activity of *Crataeva nurvala* Buch-Ham stem bark in different learning and memory paradigm viz. Elevated plus maze and Y-maze against scopolamine induced cognitive impairment. Moreover, to elucidate possible mechanism, we studied the influence of *Crataeva nurvala* ethanolic extract on central cholinergic activity via estimating the whole brain acetyl cholinesterase enzyme. Ethanolic extracts of *Crataeva nurvala* (100, 200 and 400 mg/kg body weight) were administered to adult Wistar rats for successive seven days and the acquisition, retention and retrieval of spatial recognition memory was determined against scopolamine (1 mg/kg, i.p.) induced amnesia through exteroceptive behavioral models viz. Elevated plus maze and Y-maze models. Further, whole brain acetyl cholinesterase enzyme was estimated through Ellman’s method. Pretreatment with *Crataeva nurvala* ethanolic extract significantly improved spatial learning and memory against scopolamine induced amnesia. Moreover, *Crataeva nurvala* extract decreased rat brain acetyl cholinesterase activity in a dose dependent manner and comparable to the standard drug Piracetam. The results indicate that ethanolic extract of* Crataeva nurvala* might be a useful as nootropic agent to delay the onset and reduce the severity of symptoms associated with dementia and Alzheimer’s disease. The underlying mechanism of action of its nootropic potentiality might be attributed to its anticholinesterase property.

## Introduction

Memory function is vulnerable to a variety of pathological condition including many neuropsychiatric and neurodegenerative diseases like Alzheimer’s disease (Dinesh et al., 2004[6]). Dementia, an age-related mental problem is a characteristic symptom of Alzheimer’s disease (Hanumanthachar and Milind, 2006[9]). The cholinergic hypothesis claims that decrease in cognitive function in dementia is predominantly related to a decrease in cholinergic neurotransmission. The cholinergic muscarinic antagonist like scopolamine is most widely used drug to induce amnesia in experimental animals (Kulkarni, 2013[17]). Amnesic mild cognitive impairment (through scopolamine administration) represents a transitional state between cognitive changes of normal aging and the earliest clinical features of Alzheimer’s disease (Saxena et al., 2013[23]). Nootropic drugs like Piracetam and cholinesterase inhibitors are clinically used to improve learning and memory abilities, mood and behavior in those neurodegenerative diseases (Sibi and Delphia, 2013[27]). But the resulting side-effects associated with these synthetic drugs have made their utility limited (Hanumanthachar and Milind, 2007[10]). Therefore, it is worthwhile to explore the utility of traditional medicines in the treatment of various neuropharmacological disorders associated with cognitive dysfunction as they are considered to be safe and economical (Muraleedharannair et al., 2011[18]). 

*Crataeva nurvala* (*C. nurvala*) Buch-Ham (Family: Capparidaceae) commonly known as Varuna, a well explored traditional Indian medicinal plant of Westernghats routinely used to treat various ailments in particular urolithiasis and neurological disorders (Shiddamallayya et al., 2010[25], Amod et al., 2005[1]). It is a medium sized branched deciduous plant distributed throughout the river banks of southern India and other tropical, sub-tropical countries of the world, wild or cultivated (Rajesh et al., 2011[22]). It requires dry, hot climate and shady places to grow effectively (Patil et al., 2010[21]). Hence, the present study was designed to evaluate the nootropic potentiality of *C. nurvala* through several in-vivo neuropharmacological assays and biochemical estimations. 

## Materials and Methods

### Collection and authentication of plant material

The stem bark of* C. nurvala* was collected from the stream sides of Westernghat, India and authenticated by Dr. K.V. Nagalakshamma, Professor and Head, Department of Biotechnology (UG) of St. Aloysius College, Mangalore, India. The herbarium (voucher specimen no. NGSMIPS/Hb-04/2011) was preserved in the institutional department.

### Extraction and fractionation 

**Figure 1:**
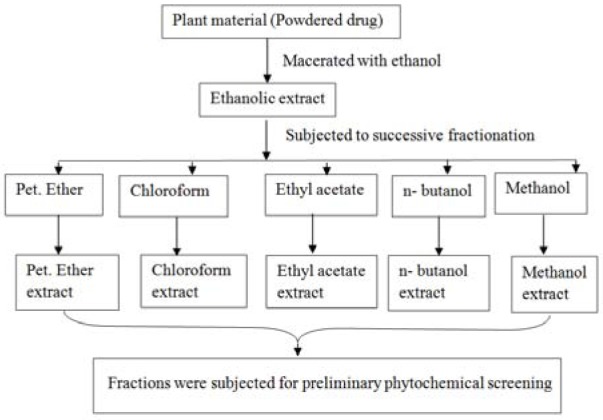
Schematic diagram of extraction and fractionation of *C. nurvala*

500 gm coarsely powdered raw material of *C. nurvala* stem bark was extracted by cold maceration with ethanol and concentrated through rotary flash evaporator at 40 °C under reduced pressure and stored in deep freezer at -20 °C (Parvin et al., 2011[20]). The yield was found to be 17 % w/w. A portion of concentrated ethanolic extract was fractionated with different solvents with increasing order of polarity (petroleum ether, chloroform, ethyl acetate, n-butanol and methanol). A flow chat of detailed method of extraction and fractionation is given in Figure 1(Fig. 1).

### Phytochemical analysis

The crude fractions were subjected to different qualitative phytochemical screening to identify the presence of various phytoconstituents as described by Harborne (Harbone, 1998[11]). The phytochemicals analyzed were alkaloids, phenols, flavonoids, saponins, terpenoids, steroids, tannins, carbohydrates, proteins and coumarins.

### Screening procedure

#### Test for tannins and phenols

5 ml of fraction was added to 2 ml of 5 % of alcoholic FeCl_3_ solution. Blue-black precipitate indicated the presence of tannins and phenols.

#### Test for alkaloids

2 ml of 2N HCl was added to 5 ml extract and heated over water bath for 10 min. The cooled solution was filtered and a few drops of Dragendorff’s reagent were added. Reddish-brown precipitate indicated the presence of alkaloid.

#### Test for saponins

About 1 g of dried powdered sample was boiled with 10 ml distilled water. Frothing persistence indicated the presence of saponins.

#### Test for terpenoids

5 ml of fraction was mixed with 2 ml of chloroform and few drops concentrated H_2_SO_4_ was carefully added to form a layer. Red ring indicated the terpenoids are present.

#### Test for steroids

5 ml of fraction was mixed with 10 ml CHCl_3_ and 1 ml acetic anhydride and few drops of concentrated H2SO4 were added. Green ring indicated the presence of steroids.

#### Test for flavonoids

To 5 ml of fraction few pieces of magnesium ribbon and few drops of concentrated HCl were carefully added. Red color indicated the presence of flavonoids.

#### Test for phlobatannins

About 2 ml of fraction was boiled with 2 ml 1 % HCl. Deposition of a red color indicated the presence of phlobatannins.

#### Test for amino acids (Ninhydrin test)

5-6 drops of ninhydrin reagent were added in 5 ml of fraction and heated over boiling water bath for 5 min. Purple coloration indicated the presence of amino acid.

#### Test for proteins (Biuret test)

5-6 drops of 5 % NaOH and 5-7 drops of 1 % CuSO_4_ were added in 2 ml fraction. Violet color indicated the presence of proteins.

### Drugs and chemicals

Scopolamine hydrobromide, piracetam, sodium carboxy methyl cellulose (Sodium-CMC), Acetylthiocholine iodide, 5,5'-Dithiobis (2-nitrobenzoic acid) (DTNB) were collected from Sigma-Aldrich, Bangalore, India. All the toxic, standard and test drugs (suspended in 0.6 % w/v of sodium CMC solution) were administered in the morning session i.e. 9 AM- 10 AM on each day.

### Acute toxicity study

The acute toxicity of ethanolic extract of *C. nurvala *stem bark was evaluated in female Wistar rats as per OECD guidelines 425 (Up and Down Procedure). A fixed dosage study was adopted for acute toxicity study where the limit dose is 2000 mg/kg body weight of test animal. Clinical signs of toxicity, body weight changes, cage side parameters and mortality rate were observed every hour for the first 6 hours and every day for 7 days (Atanu et al., 2013[3]).

### Experimental animals

Healthy young Wistar rats of either sex aged between 8-12 week old and weighing between 150-200 g obtained from the institutional animal house were used for *in-vivo* studies. The animals were housed in a room under standard environmental condition (25 °C and 50-70 % relative humidity) of 12/12 h light/dark cycle and fed with standard rat pellet and water ad libitum. Animals were allowed to acclimatize for 7 days in laboratory conditions prior to experiment (Shirish and Shrikant, 2011[26]). The experimental protocol was approved by the Institutional Animal Ethics Committee (IAEC) under the IAEC no.: KSHEMA/AEC/34/2011.

### Exteroceptive behavioral model

#### Elevated plus maze

To access the effect of *C. nurvala* on spatial long term working memory of rats Elevated plus maze test was carried out (Dinesh and Varun, 1992[7]). A typical Elevated plus maze consists of two open (50x10 cm) and two close arms (50x10x40 cm) facing each other with a central square (10x10 cm). The entire maze has been elevated to a height of 50 cm from the floor. The animals were placed individually at the end of either of open arms facing away from center and the time taken by the animals to move from open arm to enclose arm was noted on the first day (initial transfer latency, ITL). If the animal did not enter an enclosed arm within 90 seconds, it was pushed on the back into one enclosed arm and the transfer latency was given as 90 sec. Later, the animal was allowed to move freely to explore the apparatus for at least 20 sec. After the experiment, the animals were returned to their home cages and transfer latency was recorded again after 24 hours of the first exposure (retention transfer latency, RTL). The transfer latency measured on the first and second day trial served as an acquisition (learning) and retention (memory) respectively (Kasture et al., 2007[16]). From these, inflexion ration (IR) was calculated using the formula

*IR = L_0_ - L_1_ / L_1_*

where, 

IR = Inflexion ratio

L_0_ = Initial transfer latency in seconds

L_1_ = Retention transfer latency in seconds

A fall in transfer latency on subsequent maze exposures was taken as an index of successful retention (Jaiswal and Bhattarcharjee, 1992[12]).

#### Y-maze

The Y-maze task is used to measure spatial working memory through spontaneous alteration in behavior in rats. Using food as an incentive to reach the goal, animals are either required to execute a specific search sequence or minimize errors in the quest for food. Hence, temporal measurement and error scoring are the key parameters recorded for the evaluation of drug effects administered after training (Vasudevan and Milind, 2009[28]).

The Y-maze is a three-arm maze made of black painted wood with an angle of 120° between each of the two arms. Each arm is 40 cm long, 3 cm wide and 13 cm high. The three identical arms are randomly designed: start arm, in which the animal starts to explore (A); reward arm, with food stimuli (B) and other arm (C). 

Each rat was initially placed at the end of arm A, allowed to move freely and the sequence and number of arm entries were recorded manually over 8 min period. Rats tend to explore the maze systematically, entering each arm in turn. The ability to alternate required that the rats knew which arm they had already visited. The percentage of triads in which all three arms were represented, i.e., ABC, CAB, or BCA but not BAB, was recorded as an 'alternation' to estimate short-term memory. Arms were cleaned with water spray between tests to remove odors and residues. The % alternation score for each animal was defined as the ratio of the actual number of alternations to the possible number (defined as the total number of arm entries minus two) multiplied by 100 as shown by the following equation: 

% alternation = [(number of alternations) / (total arm entries - 2)] x 100

The number of arm entries was used as an indicator of locomotor activity (Se et al., 2010[24]).

### Experimental protocol

Sixty healthy Wistar rats of either sex were selected randomly and divided into ten groups of six animals each to evaluate their responses on exteroceptive behavior models. The groupings of animals were summarized below:

Group I (Normal control): Vehicle (0.6 % w/v sodium CMC) p.o.

Group II (Negative control): Scopolamine (1 mg/kg i.p.)

Group III (Positive control): Piracetam (50 mg/kg) p.o. 

Group IV: *C. nurvala* ethanolic extract (100 mg/kg) p.o.

Group V: *C. nurvala* ethanolic extract (200 mg/kg) p.o.

Group VI: *C. nurvala* ethanolic extract (400 mg/kg) p.o.

Group VII: Piracetam (50 mg/kg) p.o. + Scopolamine (1 mg/kg i.p.)

Group VIII: *C. nurvala* ethanolic extract (100 mg/kg) p.o. + Scopolamine (1 mg/kg i.p.)

Group IX: *C. nurvala* ethanolic extract (200 mg/kg) p.o. + Scopolamine (1 mg/kg i.p.)

Group X: *C. nurvala* ethanolic extract (400 mg/kg) p.o. + Scopolamine (1 mg/kg i.p.)

The dosing for all groups were done for a period of 7 days; after 45 minutes of administration of the last dose on 7^th^ day, amnesia was induced by administration of scopolamine (1 mg/kg i.p.) to Group II, VII, VIII, IX and X respectively. The negative control group (Group II) received just one dose of scopolamine (1 mg/kg i.p.) on 7^th^ day and 45 minutes after the administration of scopolamine, trials were taken on Elevated plus maze and the retention was observed after 24 hours of the first exposure i.e. on day 8 (Joshi and Parle, 2006[15]). 

The same experimental protocol was followed on the same experimental animals after one month of rehabilitation for assessment of learning and memory by Y-maze model. During experiment, the animals were exposed to food and water *ad libitum* only for 1 hour after the maze exposure for the day to ensure motivation towards reward area (B) (Saxena et al., 2013[23]).

### Biochemical estimation 

#### Collection of brain sample

Immediately after the experiment animals were sacrificed by cervical decapitation under light anesthesia and whole brain was carefully removed from the skull. The fresh whole brain was weighed, kept on ice bath, rinsed with ice-cold isotonic saline and homogenized (approximately 20 mg of tissue/ml of phosphate buffer (pH 8.0; 0.1M) in a Potter-Elvehjem homogenizer. The homogenate was centrifuged at 3000 rpm for 10 min and the resultant cloudy supernatant liquid was used for the estimation of AchE activity (Vinutha et al., 2007[30]). 

#### Estimation of brain acetyl cholinesterase (AChE) activity

The AchE activity was assessed by Ellman's method (Ellman, 1959[8]). The AchE activity is measured by using an artificial substrate Acetyl thiocholine (ATC). AchE acts on ATC and release thiocholine and acetic acid. Thiocholine further is allowed to react with -SH reagent 5,5-dithio-bis- (2, nitro benzoic acid) (DTNB) which is reduced to thionitro benzoic acid, a yellow coloured anion with absorption maxima 412 nm. The molar extinction coefficient of thionitro benzoic acid is 1.36x10^-4^/molar/cm. The concentration of thionitro benzoic acid is determined using Shimadzu UV-1700 Pharmac-spec UV-Vis spectrophotometer which is directly proportional to AchE activity. The rate of the reaction was calculated using following formula 

*R = 5.74 x 10^-4^ x ∆A/C_O_*

where,

R = rate in moles of substrate hydrolyzed / minute / gm tissue

∆A = change in absorbance / min 

C_O_ = original concentration of the tissue (mg/ml) 

### Statistical significance

The results of *in-vivo* studies were expressed as mean ± SEM (standard error of mean). The difference between the control and treated means were analyzed using one way analysis of variance (ANOVA). P-values < 0.05 were taken to be statistically significant. Tukey's test was used for multiple comparisons between all columns. The statistical analysis was done using the software Graph-pad prism version no: 5.0. 

## Results

### Phytochemical analysis

Qualitative estimation revealed that stem bark extract of *C. nurvala* were enriched with alkaloids, phenolic compounds and tannins, phytosterols and triterpene, flavanoids, saponins, and coumarins. The detailed summary of phytochemical screening of *C. nurvala* fractions is shown below (Table 1(Tab. 1)).

### Acute toxicity study

No mortality was observed following oral administration of *C. nurvala* ethanolic extract even with the highest dose (2000 mg/kg). Moreover, no significant changes in body weight and behavior were observed. Hence, *C. nurvala* could be safe up to the dose of 2000 mg/kg body weight of the animal.

### Elevated plus maze

**Figure 2:**
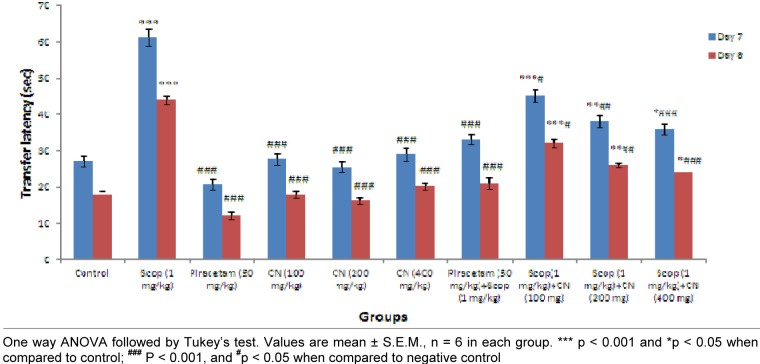
Effect of *C. nurvala* extract on transfer latency (in seconds) on Elevated plus maze

Results suggest pre-treatment with ethanolic extract of *C. nurvala* (100, 200 and 400 mg/kg body weight) for seven successive days did not exhibited much difference in ITL compared to normal control group but in presence of amnesia, higher dose of *C. nurvala* ethanolic extract (400 mg/kg body weight) afforded a significant (**^###^**p<0.001) decrease in TL (score: 35.83 ± 1.537) compared to other lower doses groups (score: 45.16 ± 1.759 and 38.00 ± 1.693 respectively) and closely approximated to standard drug Piracetam (score: 33.16 ± 1.558). However, all the three doses of extract showed improvement spatial learning and memory activity in dose dependant manner. Moreover, in Group VIII, IX and X, significant decrease in RTL on Day 8 compared to Day 7 were observed which elaborate the drugs responses to overcome the learning and memory deficits produced by scopolamine. Further, significant improvements in inflexion ratio in the treatment groups suggest its nootropic potential (Figure 2(Fig. 2)).

### Y-maze

Y-maze test was performed to measure the effect of *C. nurvala* extract on spatial recognition memory of on Wistar rat model. The effect on alteration behavior was studied based on two parameters viz. % alteration and no. of arm entries.

#### Effect on % alteration

**Figure 3:**
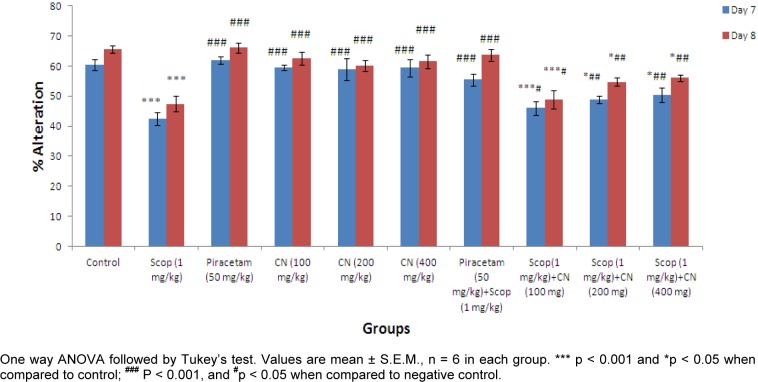
Effect of *C. nurvala* extract on % Alteration behavior in Y-maze

Pre-treatment with ethanolic extract of *C. nurvala* (100, 200 and 400 mg/kg body weight) did not exhibit much difference in alteration response in comparison to normal control group but in presence of amnesia, higher dose of *C. nurvala* ethanolic extract (400 mg/kg body weight) afforded significant (**^###^**p 0.001) alteration response (score: 50.35 ± 2.364) compared to other lower doses groups (score: 45.98 ± 2.326 and 48.89 ± 1.185 respectively) and closely approximated to standard drug Piracetam (score: 55.46 ± 2.062). However, all the three doses of extract showed improvement in % alteration in dose dependant manner. Moreover, in Group VIII, IX and X, significant increase in % alteration on Day 8 compared to Day 7 were observed (Figure 3(Fig. 3)).

#### Effect on number of arm entries

**Figure 4:**
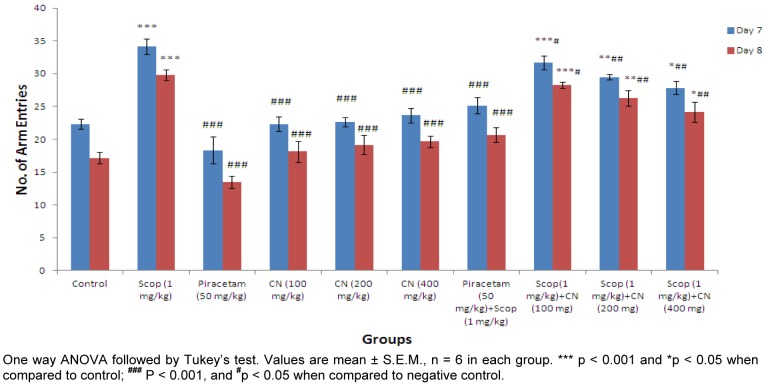
Effect of *C. nurvala* on number of arm entries in Y-maze

Higher dose of *C. nurvala* ethanolic extract (400 mg/kg body weight) afforded significant (**^###^**p 0.001) decline in number of arm entries (score: 27.83 ± 0.980) compared to other lower doses groups (score: 31.67 ± 1.085 and 29.50 ± 0.428 respectively) and closely approximated to standard drug Piracetam (score: 25.16 ± 1.249). However, all the three doses of extract showed decrease in number of arm entries in dose dependant manner. Moreover, in Group VIII, IX and X significant decrease in number of arm entries on Day 8 compared to Day 7 were observed (Figure 4(Fig. 4)).

#### Estimation of whole brain acetylcholine esterase (AchE) activity

**Figure 5:**
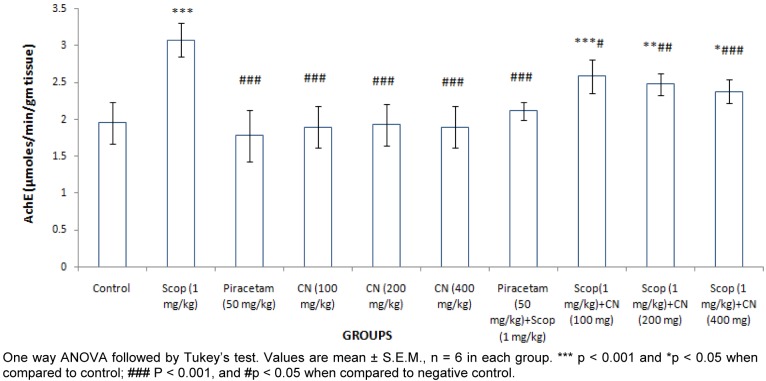
Effect of *C. nurvala* extract on whole brain acetyl cholinesterase enzyme

The acetylcholiesterase activity of whole brain was significantly (***p < 0.001) elevated after scopolamine (1 mg/kg, i.p.) treatment. Pre-treatment with Piracetam (50 mg/ kg, p.o.) and *C. nurvala* (100, 200 and 400 mg/kg, p.o.) significantly (**^###^**p < 0.001) lowered AChE activity (Figure 5(Fig. 5)).

## Discussion

Cognition is a process of storing sequence of information in a systematic manner by which one become aware of their surroundings, objects and thoughts (Ashutosh et al., 2002[2]). Cognitive impairment is one of the major health problems in normal aged life as well as in some neurological disease conditions like Alzheimer's (Vasudevan and Milind, 2007[29]). Herbal cognition enhancers can be used as an alternative to facilitate attention abilities and to attenuate the impairment of cognitive functions associated with age and age-related pathologies (Oguz et al., 2011[19]). Since, no study had been conducted to evaluate the nootropic potential of stem bark of *C. nurvala*, the present study was conducted.

The phytochemical screening revealed stem bark fractions of *C. nurvala* were enriched with different secondary metabolites in their respective fractions. Further, various bioactive phytoconstituents viz. β-sitosterol, stigmasterol, melatonin, lupeol, catechin had been isolated from the stem bark of *C. nurvala* extract (Atanu et al., 2013[5], 2014[4]). Melatonin is a potent anticholinesterase and antioxidant agent which might be associated with the nootropic activity of *C. nurvala* extract (Jian-zhi and Ze-fen, 2006[14]; Jemima et al., 2011[13]). 

The findings from Elevated plus maze test suggest *C. nurvala* ethanolic extract showed protective effect in transfer latency against scopolamine induced amnesia in dose dependent manner. Moreover, the decrease in transfer latency during retention period suggested the drug response to overcome learning and memory deficit produced by scopolamine. Further, nootropic activity was evaluated through Y-maze test and results suggest an improvement in % alteration and decrease in number of arm entries against scopolamine induced amnesia in dose dependent manner. These provide an adequate scientific promise to validate the nootropic potency of *C. nurvala* extract. 

Moreover, *C. nurvala* extract significantly inhibit whole brain AchE against scopolamine induced amnesia in dose dependent manner and thereby could increase the availability of acetylcholine in brain and in cholinergic synapse. These observations postulated potent anticholinesterase activity of *C. nurvala* extract which might be one of the possible mechanisms to encounter with cognitive disorders.

Therefore, in the present study we observed that *C. nurvala *extract

1. showed improvement in memory of Wistar rats when tested on exteroceptive behavioral model viz. elevated plus maze and Y-maze
2. inhibited whole brain acetylcholinesterase enzyme activity. Thus, a combination of anticholinesterase and neuroprotective effects exhibited by *C. nurvala *extract may be responsible for its nootropic potentiality.

However, investigations using more experimental paradigms particularly with isolated bioactive compounds like melatonin may be required for further confirmation of nootropic potential of *C. nurvala *stem bark in the treatment of Alzheimer's and other cognitive disorders. 

## Conflict of interest

We declare that we don’t have any conflict of interest.

## Acknowledgements

The authors acknowledge the financial support of Nitte University, Mangalore, India for the research work (Grant no. NU/PhD/Pharm/Res-10/2011).

## Figures and Tables

**Table 1:**
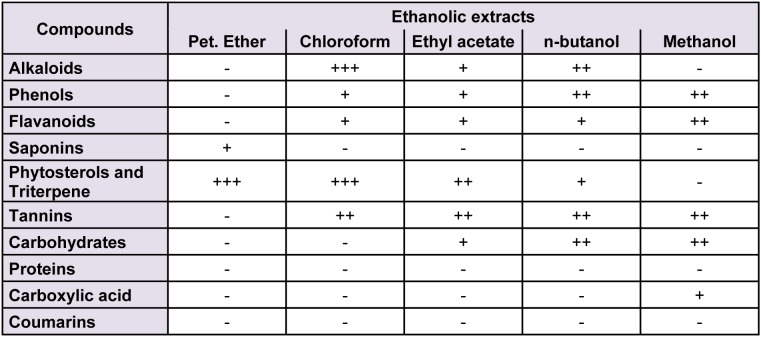
Preliminary phytochemical screening of *C. nurvala*
